# Society Meetings

**Published:** 1908-04

**Authors:** 


### SOCIETY MEETINGS.

The American Medico-Physiological Association will hold its sixty-fourth annual meeting at Cincinnati, May 12-15, 1908, under the presidency of Dr. Charles P. Bancroft, of Concord, N. H. The headquarters of the association will be at the Hotel Sinton. Dr. Charles W. Pilgrim, of Poughkeepsie, is the secretary.

The Buffalo Academy of Medicine held meetings during March as follows:

*Section on Surgery.*—Tuesday evening, March 3. Program: (1) Demonstration of direct bronchoscophy and esophagoscopy, F. Whitehill Hinkel; (2) Reports of cases of ecchinoccus cyst of the liver, Marshall Clinton, Herman E. Hayd; (3) Spinal sprain; its complications and consequences; with report of 26 cases, Prescott Le Breton; (4) Flat foot, Roland Meisenbach.

*Section on Medicine.*—Tuesday evening, March 10. Program: (*a*) A case of paranoia, Sidney A. Dunham; (*b*) Specific infections arthritis, J. Henry Dowd.

*Section on Pathology.*—Tuesday evening, March 17. Program: (*a*) Report of a case of gangrenous inflammation of the appendix, with spontaneous discharge into the small intestines, A. L. Benedict; (*b*) Epithelial tumors of the skin and exposed mucous membrane. Lantern slides, A. H. McGlannan, associate in surgery, college of physicians and surgeons, Baltimore.

*A Stated Meeting* was held Tuesday evening, March 24. The program was furnished by the Section on Obstetrics and Gynecology, as follows: (*a*) Transverse abdominal incision in pelvic surgery, its advantages and limitations, James E. King; (*b*) Treatment of diffused or spreading peritonitis, Frank McGuire.

---

The Rochester Academy of Medicine held meetings during the month of March as follows:

*Section on General Medicine.*—Wednesday evening, March 4. Program: Acute anterior poliomyelitis, Robert G. Cook; Tubercular hip disease, occurring in the aged, with presentation of case, L. A. Whitney.

*Section on Surgery.*—Wednesday evening, March 11. Program: General observations and conclusions after a short visit to the Drs. Mayo, Drs. Zimmer and Elsner.

*Section on Obstetrics, Gynecology, and Pediatrics.*—Wednesday evening, March 18. Program: (1) Report of a case of chorea at term; (2) (3) Report of two cases of eclampsia; (4) Report of an alleged case of abortion, C. S. Starr.

*Section on Public Health.*—Wednesday evening, March 25. Program: Investigation of medico legal cases. The nervous examination, E. B. Angell; The surgical aspect, E. W. Mulligan; The detection of malingering, W. J. Herriman. (By invitation.)

The Committee on Legislation of the Medical Society of the County of New York, voted unanimously to actively support senate bill No. 636, and assembly bill No. 1215, and requested its chairman, E. Eliot Harris, to prepare suitable resolutions to be submitted to the society for its action. The following were unanimously adopted at the regular meeting of the society held March 23, 1908:

Whereas the commission, appointed by Governor Hughes pursuant to an act of the last legislature, has investigated and reported on a suitable site for the Eastern New York State Custodial Asylum and has officially published that there are at least 20,000 feeble-minded and epileptic persons in this state; that the existing State Custodial Institutions located at Rome, Syracuse, Newark and Sonyea are now overcrowded; that they house 3,250 persons while their capacity is authoritatively reported for only 3,183 inmates, making an excess of 67 persons which added to 1,000 applicants who are now on the waiting list of these institutions and 1,808 patients improperly confined in almhouses of the state, make a total of 2,875 patients needing immediate state custodial care—to say nothing of the much larger number of the 20,000 persons in private homes who require state custodial care for the protection of themselves and for the protection of the best interests of the commonwealth.

Whereas the site selected is admirably suited for the purposes of the colony as shown in the report of Mr. Kuichling, the consulting engineer: the options on the property were secured at a time which gives the state a remarkable opportunity to be possessed of a tract of land at the lowest possible cost: its value will rapidly enhance with the improvement in the financial conditions of the county: that the proposed colony will be located near Haverstraw, Rockland County, 22 miles north of New York City, is a great blessing to these unfortunate poor as well as being of great economic value to the state, as it may be readily and cheaply reached by train or boat: the round trip being $1.00 by train and much less by boat: that more than 50 per cent. of the inmates and over 61 per cent. of those on the waiting lists of the distant state institutions are from the vicinity of Greater New York accounts for the many instances where it seems cruel to send these poor patients so far from their family relations and friends, who plead pitifully for their retention in the city institutions which are accessible to them, for they know full well that the time and the expense of the journey will in most cases prohibit them from ever seeing their near ones again, which becomes evident when we consider that the round-trip railroad fare alone is $14.00 to the Craig Colony for Epileptics at Sonyea—the present cost to the department of public charities of this city to send a patient with an attendant to the colony is over $25.00 dollars. If a colony were established at Haverstraw then the regular boat of the department could make the trip easily and without cost to the visitors entitled to permits.

Therefore be it

*Resolved,* That this society earnestly requests the finance committee of the senate to report favorably Senator Armstrong's bill No. 636, which provides for an appropriation of $188,575.00 for the site near Haverstraw, Rockland County. The members of the senate are also urgently requested to vote for the final passage of the bill which has already passed the assembly unanimously as assembly bill No. 1215.

*Resolved,* That the Committee on Legislation be empowered to use these resolutions in coöperating with other committees working for the enactment of the bill to purchase the site of the Eastern New York State Custodial Asylum.

---

The American Proctologic Society will hold its tenth annual meeting at Chicago, June 1 and 2, 1908, under the presidency of A. Bennett Cooke, of Nashville. The headquarters and place of meeting will be at the Palmer House. Lewis H. Adler, Jr., of Philadelphia is the secretary.

The profession is cordially invited to attend all meetings.

---

The American Gastro-Enterological Association will hold its eleventh annual meeting at Chicago, June 1 and 2, 1908, under the presidency of J. P. Sawyer, of Cleveland.

